# Glycolytic pathway candidate markers in the prognosis of oral squamous cell carcinoma: a systematic review with meta-analysis

**DOI:** 10.1590/1414-431X202010504

**Published:** 2021-01-25

**Authors:** S.E.C. de Mattos, L.F. Diel, L.S. Bittencourt, C.E. Schnorr, F.A. Gonçalves, L. Bernardi, M.L. Lamers

**Affiliations:** 1 Programa de Pós-graduação em Ciências Biológicas, Fisiologia, Universidade Federal do Rio Grande do Sul, Porto Alegre, RS, Brasil; 2 Faculdade de Odontologia, Universidade Federal do Rio Grande do Sul, Porto Alegre, RS, Brasil; 3 Departamento de Ciências Morfológicas, Instituto Básico de Ciências da Saúde, Universidade Federal do Rio Grande do Sul, Porto Alegre, RS, Brasil; 4 Instituto Federal da Educação, Ciência e Tecnologia do Rio Grande do Sul - Porto Alegre Campus, Porto Alegre, RS, Brasil; 5 Secretaria de Educação do Estado do Rio Grande do Sul, Escola Técnica em Saúde, Hospital de Clínicas de Porto Alegre, Porto Alegre, RS, Brasil; 6 Departamento de Ciências Naturales y Exactas, Universidad De La Costa, Barranquilla, Atlántico, Colombia

**Keywords:** Oral cancer, Survival, Prognosis, Disease-free survival, Energy metabolism

## Abstract

Molecular changes that affect mitochondrial glycolysis have been associated with the maintenance of tumor cells. Some metabolic factors have already been described as predictors of disease severity and outcomes. This systematic review was conducted to answer the question: Is the glycolytic pathway correlated with the prognosis of oral squamous cell carcinoma (OSCC)? A search strategy was developed to retrieve studies in English from PubMed, Scopus, and ISI Web of Science using keywords related to squamous cell carcinoma, survival, and glycolytic pathway, with no restriction of publication date. The search retrieved 1273 publications. After the titles and abstracts were analyzed, 27 studies met inclusion criteria. Studies were divided into groups according to two subtopics, glycolytic pathways and diagnosis, which describe the glycolytic profile of OSCC tumors. Several components of tumor energy metabolism found in this review are important predictors of survival of patients with OSCC.

## Introduction

Two decades ago, cellular glucose metabolism and cancer metabolism were not major branches of cancer biology research. However, in the past 15 years, there has been a growing interest in cancer metabolism, particularly the energy metabolism of cancer cells. These topics have now become an integral part of cancer biology, as are signal transduction and transcription (1).

Cancer is characterized by a disorganized tissue growth and the presence of tumor cells that are capable of invading adjacent tissues and traveling through blood and lymphatic vessels to form metastases (2). Oral squamous cell carcinoma (OSCC) is the eighth most prevalent cancer worldwide, with more than 300,000 new cases diagnosed every year. It is a public health problem because of its poor prognosis, with a 5-year survival rate below 50% (3).

An OSCC prognosis is conventionally based on the clinical Tumor, Nodes, Metastasis (TNM) classification. However, this system does not predict patient survival at the time of initial diagnosis. More sensitive prognostic biomarkers may be useful to define patient follow-up and treatment in the first stages of tumor development (4). Because of that, the number of studies about metabolic factors that promote tumor development and metastasis has increased.

Molecular changes that affect glycolysis are associated with the maintenance of the capacity of a tumor cell to survive, proliferate, and invade tissues when exposed to adverse conditions, such as hypoxia, lack of nutrients, and immune responses (5,6).

Some metabolism factors, already described as predictors of disease severity and outcome, are metabolic biomarkers that may be useful in identifying OSCC relapse in low- and high-risk patients (7–9). This systematic review used a search strategy to retrieve studies to answer the research question: Is the glycolytic pathway correlated with the prognosis of OSCC?

## Material and Methods

A search of the electronic databases PubMed, Scopus, and ISI Web of Science used keywords related to oral squamous cell carcinoma, survival, and energy metabolism to retrieve studies from the literature. The search strategy to retrieve studies according to titles and abstracts was: “head and neck neoplasms” OR “mouth neoplasms” OR “oral squamous cell carcinoma” OR “head and neck squamous cell carcinoma” OR “head and neck cancer” OR “oral cancer” AND “survival” OR “mortality” OR “prognosis” OR “disease free survival” OR “survival analysis” AND “energetic metabolism” OR “energy metabolism” OR “glucose metabolism” OR “mitochondrial metabolism” OR “ATP production” OR “oxidative phosphorylation” OR “positron emission tomography” OR “glucose transporters”. Only studies in English were included, with no date restriction (last access May 2020), and duplicate studies were discarded. After initial screening, studies were evaluated according to inclusion and exclusion criteria by two reviewers (S.E.C.M. and F.A.G.). If the reviewers disagreed, the study was evaluated again until a consensus was reached. Study authors were not contacted to obtain any additional information. This systematic review and meta-analysis was registered with PROSPERO: CRD42018106978.

### Inclusion and exclusion criteria

Only studies about the effect of glycolytic metabolism on the survival of human patients with OSCC were included. Studies using only animal models or cell cultures, case reports, and literature reviews were excluded after titles and abstracts were read. Studies were also excluded if they did not include a survival analysis or follow-up time, if they included patients that had not finished treatment, or if their focus was on something other than glycolytic variables. The following inclusion criteria were used in this meta-analysis: 1) the publication explored the relationship between GLUT1/TKTL1 expression and high SUVmax and OSCC prognosis, such as overall survival (OS); 2) the expression of GLUT1/TKTL1 and 2-deoxy-2-[fluorine-18]fluoro-D-glucose (FDG) uptake was detected in tumor tissue; 3) the expression of GLUT1/TKTL1 was measured by the standard methods of immunohistochemistry and RT-PCR with the corresponding cut-off value; 4) there were sufficient, clear, and available data to extract or estimate hazard ratios (HR) and 95% confidence interval (CI); 5) each study had a size greater than twenty individuals; studies with small n could impair precision through increasing heterogeneity; 6) studies were published in English; and 7) the meta-analysis was restricted to original articles (no expert opinions, editorials, or reviews).

### Statistical analysis

The software Comprehensive Meta-Analysis version 3 (downloaded from http://www.meta-analysis.com) was employed to integrate and analyze data.

The effect size is reported as HR and the corresponding 95%CI. A pooled HR >1 suggested a poor prognosis of patients with GLUT1/TKTL1 high-expression and high SUVmax, whereas HR <1 entailed a better one. I^2^ and Q-test indicated the degree of inconsistency across the included trails, I^2^>50% and P<0.05 indicated uncompromising heterogeneity (10,11). Fixed effects model or random-effects model was chosen for the low or high heterogeneity analysis, respectively. Sensitivity analysis was conducted via excluding low-quality studies and interchanging random effect model and fixed effect model among included trials to ensure the stability of pooled data. Moreover, Egger’s weighted regression test and Begg’s rank correlation test were applied to scrutinize publication bias amongst included studies. A P-value <0.05 indicated statistical significance. All analyses adopted in this article were totally based on previously published studies; therefore, no ethical approval and consent from patients were required.

## Results

**Figure 1:**
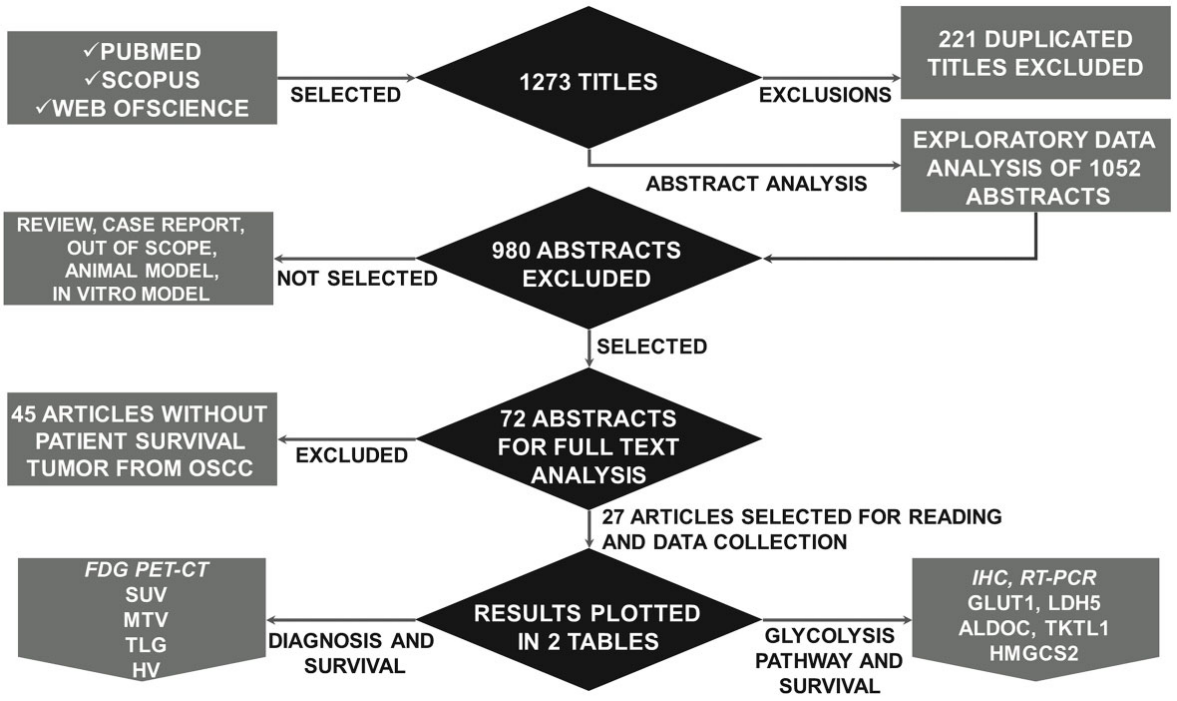
Study flow diagram for the literature search and selection of articles for this systematic review and meta-analysis.

The initial search retrieved 1273 studies. After 221 duplicates were discarded, an evaluation of titles and abstracts resulted in 72 studies for full-text reading, after which 45 other studies were excluded (Figure 1). This review, therefore, included 27 studies. The studies included were classified according to two subtopics: i) glycolysis pathway and ii) diagnosis. These subtopics describe the metabolic profile of OSCC tumors. Supplementary Tables S1 and S2 provide information about study selection.

### Glycolytic pathway

Eleven studies evaluated markers related to glycolysis pathway: glucose transporter 1 (GLUT1), transketolase-like 1 (TKTL1B), L-lactate dehydrogenase B chain (LDHB), lactate dehydrogenase 5 (LDH5), pyruvate kinase (PKM2), fructose-bisphosphate aldolase C expression (AldoC), carbonic anhydrase 9 (CA9), mitochondrial 3-hydroxy-3-methylglutaryl-CoA synthase (HMGCS2), and binding protein Apo10 (Apo10) (12–22 [Bibr B23]). Five studies (13,14,16,18,21) used immunohistochemistry (IHC) to examine the correlation of GLUT1 expression levels with prognosis. Low and high GLUT1 expression levels were analyzed using univariate and multivariate tests. In all studies, GLUT-1 expression was significantly correlated with disease-specific survival, and higher protein levels were associated with worse prognosis and shorter median survivals.

Five studies (13,15,17,19,20) described the enzymes AldoC, CA9, PKM2, LDHB, and LDH5. Low and high levels of AldoC mRNA and protein expression, determined by IHC, correlated with survival. High expression levels of PKM2, LDHB, and LDH5 were significantly associated with poorer survival, while patients with tumors expressing low AldoC mRNA had longer survivals (P=0.001). Also, OSCC patients with low HMGCS2 mRNA (12) and Apo10 and TKTL1B (22) protein expression survived significantly longer than those with high expression levels. Results suggested that these metabolic factors related to the glycolysis pathway may play a key role in oral carcinoma progression.

### GLUT-1 expression and OSCC overall survival

**Figure 2:**
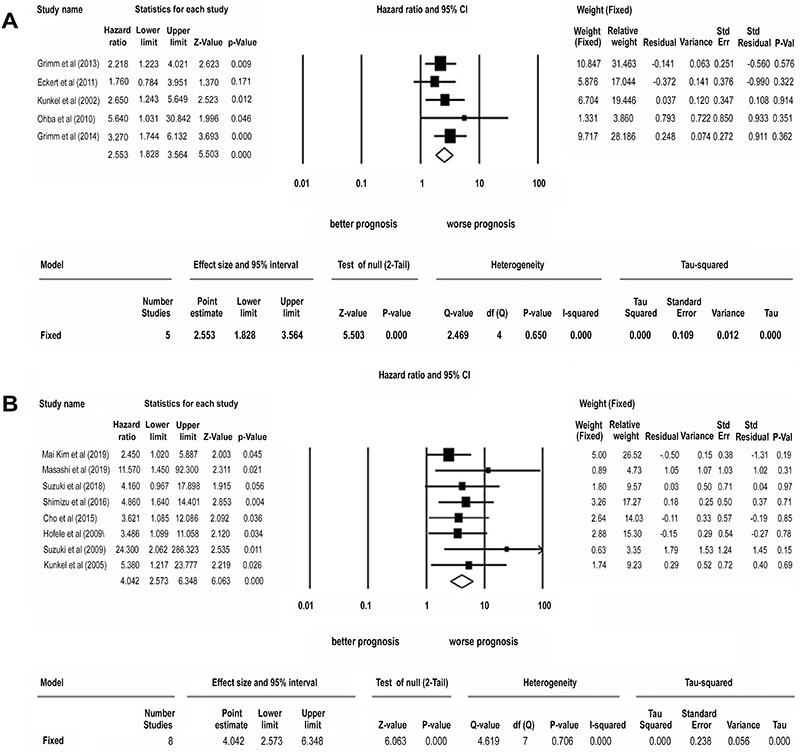
A, Correlation between GLUT-1 and overall survival in oral squamous cell carcinoma (OSCC) (HR=2.553, 95%CI: 1.828-3.564, P=0.000, fixed effect model). **B**, Correlation between positron emission tomography-computed tomography with 2-deoxy-2-[fluorine-18]fluoro-D-glucose (FDG) standardized uptake value-max parameter and overall survival in OSCC (HR=4.042, 95%CI: 2.573-6.348, P=0.000, fixed effect model). GLUT-1: glucose transporter-1; HR: hazard ratio. See Supplementary Table S1 for the reference numbers of the studies.

**Figure 3:**
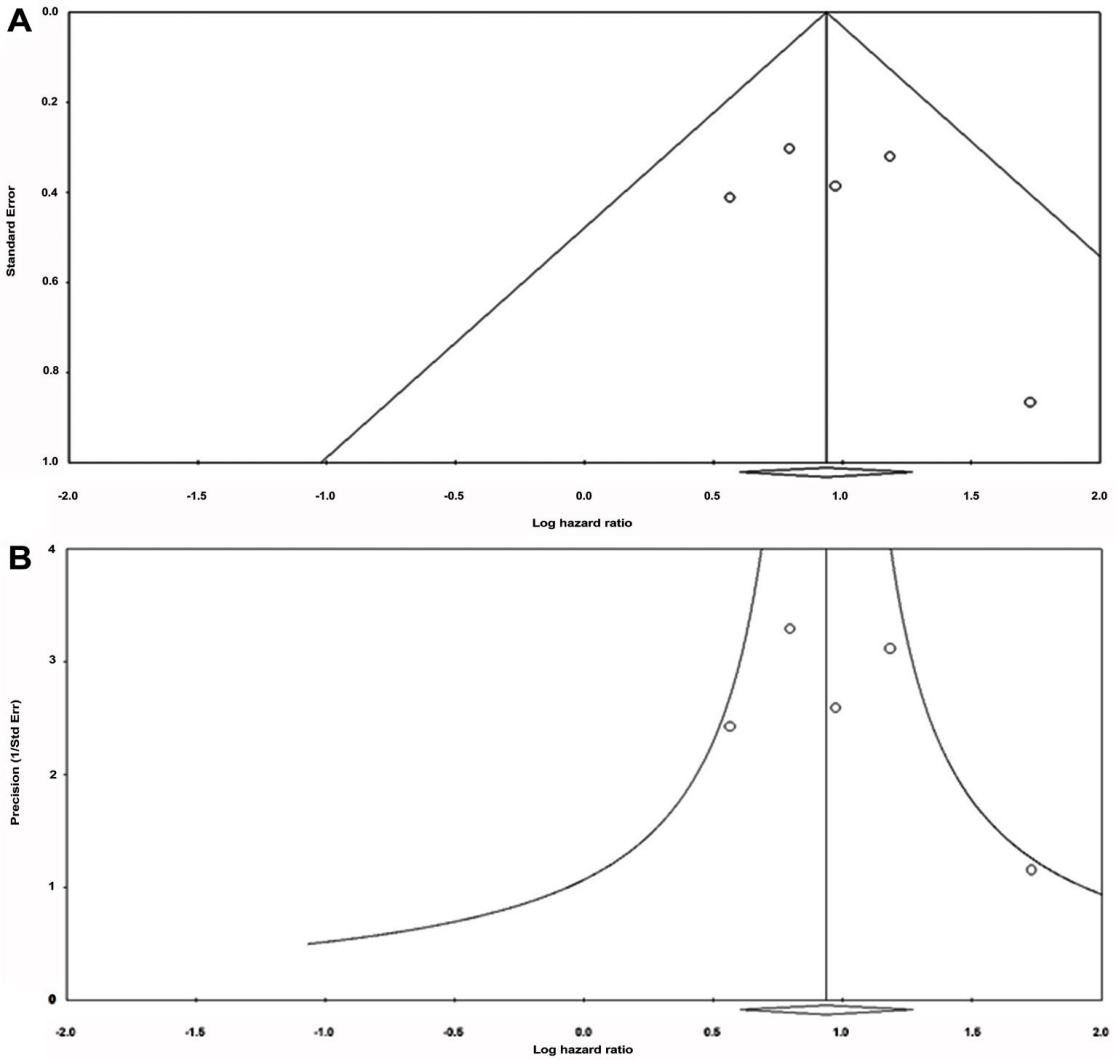
Publication bias assessment for glucose transporter-1 (GLUT-1) and overall survival. **A**, Begg’s test (funnel plot) and **B**, Egger’s test (precision plot).

The combined analysis of 5 studies (13,14,16,18,21) showed that high GLUT-1 expression was related to worse OS (pooled HR=2.927, 95%CI: 2.121-4.041, z-value=6.530 P=0.000) (Figure 2A). Due to the non-significant heterogeneity among included studies (I^2^=0.000 P= 0.650), a fixed effect model was performed to pool HRs. The results for Begg’s test and Egger’s test revealed that there was no significant publication bias for all analyses (Table 1; Figure 3).

**Table 1:** Publication bias tested by Begg’s test and Egger’s test in meta-analysis.

Variables	No. of studies	Begg’s rankcorrelation P-value	Egger’s regression intercept P-value
GLUT-1 and OS	5	0.80650	0.48721
SUVMax and OS	8	0.00937	0.00085

OS: overall survival.

### Diagnosis

Positron emission tomography-computed tomography (PET-CT) with FDG is a diagnostic tool that uses glucose cell absorption to identify tumor volume and localization. PET-CT uses parameters such as the standardized uptake value (SUV), hypoxic volume (HV), metabolic tumor volume (MTV), and total lesion glycolysis (TLG). Fifteen of the studies in this review reported data about FDG PET-CT findings and patient survival. One of these studies has already been mentioned in the analysis of studies about GLUT1. These studies used different values of SUV to classify OSCC tumors according to low or high SUVmax levels (16,). Regardless of subgroup or protocol, all these studies found that high levels of SUV before surgery or follow-up predicted adverse outcomes for patients with OSCC. Using the same tool, eight studies evaluated MTV and TLG alone or in combination with SUVmax (24,25 [Bibr B26] [Bibr B27] [Bibr B28] [Bibr B29],^30^ [Bibr B31] [Bibr B32] [Bibr B33],^34^–38), and some found that both MTV and TLG might be prognostic for the survival of patients with oral cancer.

**Figure 4:**
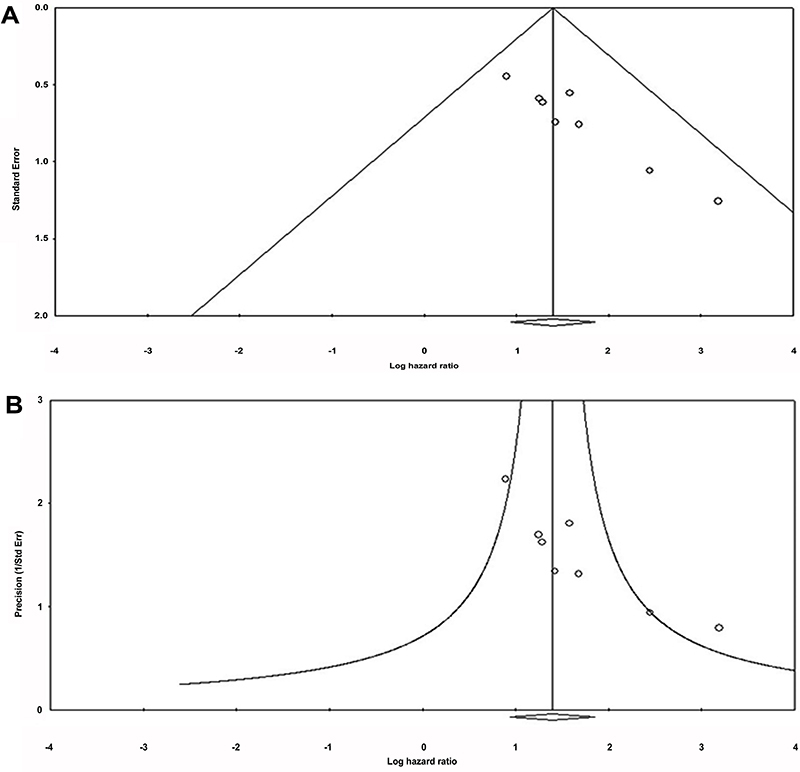
Publication bias assessment for positron emission tomography-computed tomography standardized uptake value-max and overall survival. **A**, Begg’s test (funnel plot) and **B**, Egger’s test (precision plot).

For meta-analysis we included the data of 8 combined studies (25,27,29,31,33,34,37,38). The high SUVmax parameter was related to worse OS in OSCC (pooled HR=4.042, 95%CI: 2.573-6.348, z-value=6.063, P=0.000) (Figure 2B). Due to the low heterogeneity among included studies (I^2^=0.000 P=0.706), a fixed effect model was performed to pool HRs. The results for Begg’s test and Egger’s test revealed that there was significant publication bias (Table 1; Figure 4).

## Discussion

Molecular oral cancer changes result in lesions that develop in a series of histopathologic stages (39,40). Some of these molecular mechanisms are involved in tumor metabolism. Our review focused on the literature about the impact of tumor glycolytic factors on the survival of patients with OSCC.

Numerous studies have investigated metabolic changes in cancer, but this review found only 27 studies that met our inclusion criteria and discussed the role of metabolism in the prognosis and survival of patients with oral cancer. Ten studies evaluated specific markers of the glycolysis pathway, and ten others used PET-CT as a diagnostic method. One study used both the GLUT1 marker and PET-CT. The glycolytic enzymes PKM2, PDK1, and HK2 are greatly important in the functionality of glycolytic and mitochondrial activity, and the studies included here confirmed the positive regulation of oxygen-dependent and oxygen-independent energy production.

**Figure 5:**
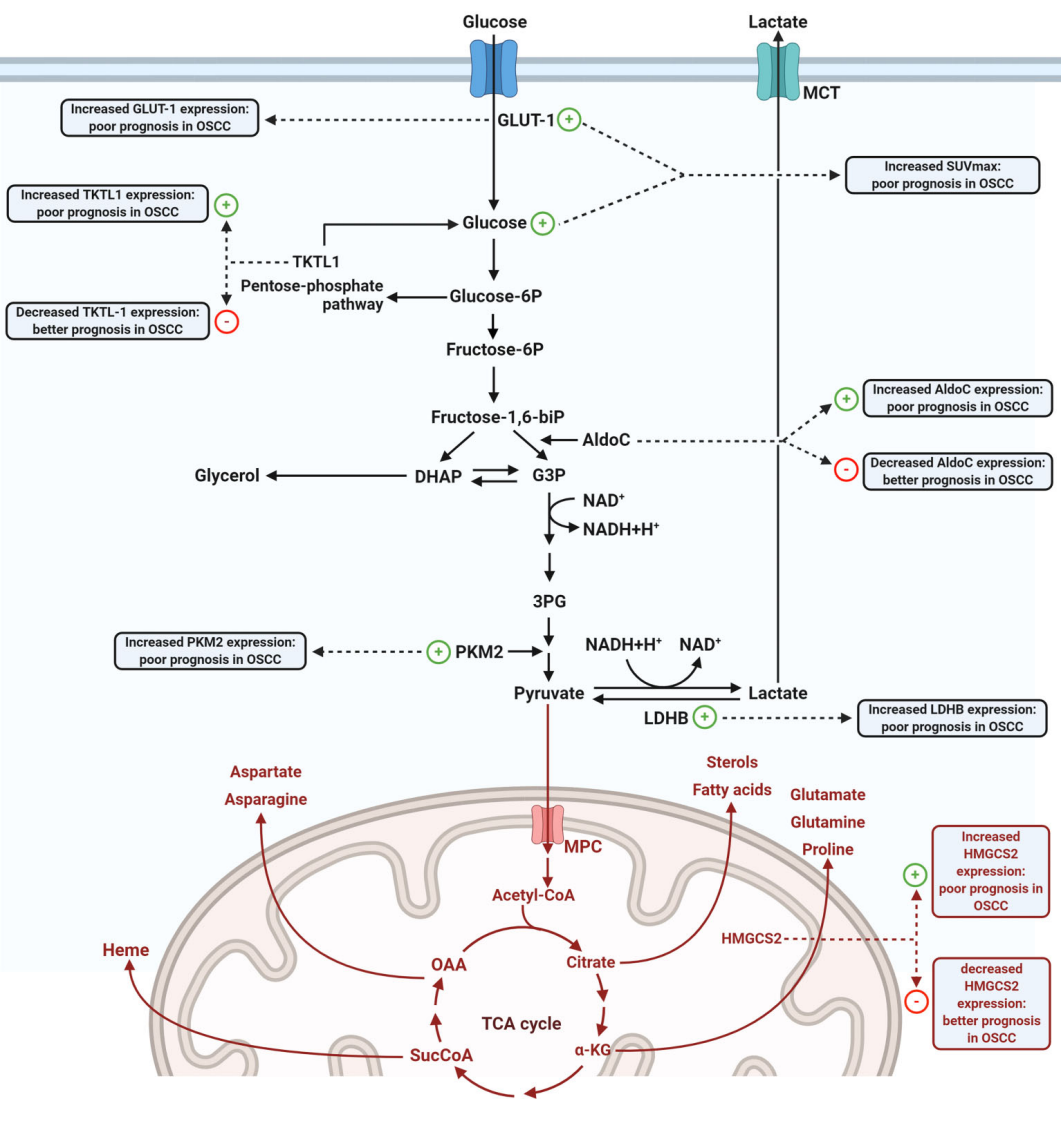
Flow diagram showing the impact of each marker on the energy metabolism of tumor cells evaluated by meta-analysis. The + and - symbols indicate increase and decrease of marker expression, respectively. GLUT-1: glucose transporter 1; TKTL-1: transketolase like-1; SUVmax: standard uptake value maximum; AldoC: fructose-bisphosphate aldolase C; PKM2: pyruvate kinase isoform M2; HMGCS2: mitochondrial hydroxymethylglutaryl-CoA synthase; LDHB: lactate dehydrogenase B; OSCC: oral squamous cell carcinoma; TCA: tricarboxylic acid; OAA: oxalacetate acid; MCP: mitochondrial pyruvate carrier; MCT: monocarboxylate transporter.

All glycolytic pathway markers expressed in tumor cells and included in this review were associated with poorer patient prognosis (Figure 5). GLUT-1 high expression was significantly correlated with shorter OS (Figure 2A) and these results are similar to a meta-analysis of (41), which specifically evaluated the GLUT-1 expression in OSCC and its correlation with shorter OS. Cancer cells produce ATP via glycolysis mainly, and not through the tricarboxylic acid cycle or oxidative phosphorylation. Consequently, large amounts of glucose are transported into the cytoplasm to maintain high levels of ATP production. Cancer cells express high levels of glucose transporters in the cytoplasmic membrane, particularly GLUT1 and GLUT3 (42,43). GLUT-1 possesses high affinity and provides potential energy for cellular growth. Overexpression of GLUT-1 could facilitate growth and proliferation of tumor cells through supporting the high metabolic glucose consumption in the hypoxic tumor microenvironment (TME), which means that changes of GLUT-1 levels can be influenced by growth rate, oxygen supply, and malignant transformation in TME (41).

This increase in the expression of glucose transporters is affected by HIF-1, which also increases the expression of the genes involved in the enzymatic breakdown of glucose into pyruvate and of the enzymes involved in pyruvate metabolism. In hypoxic cells, pyruvate is converted into lactate by lactate dehydrogenase (LDH). However, in cancer cells, the intense transformation of pyruvate into lactate persists with or without subsequent exposure to oxygen, a historical phenomenon known as the Warburg effect (44). According to Sun et al. (19) and Grimm et al. (13), a high level of LDH is a predictor of poor survival in patients with OSCC (13,19). LDH may contribute to the increase of the lactate released into the extracellular space and decrease pH levels in the tumor environment, promoting cancer migration, invasion, and metastasis (45).

Lactate production is directly affected by pyruvate levels. Pyruvate is converted from phosphoenolpyruvate by pyruvate kinase, mainly through its isoform, PKM2, which is expressed in both cancer and normal tissues and seems to promote anabolic metabolism and the Warburg effect (46,47). Additionally, the nuclear translocation of PKM2 acts as a transcriptional factor that mediates epithelial-mesenchymal transition (EMT) in colon cancer cells (48). PKM2 overexpression is correlated with a poor overall survival in oral, gastric, and bladder cancer (49,50). Inflammation is controlled by several extracellular mediators and regulators including cytokines, growth factors, and eicosanoids. The study of the impact of these molecules (mainly interleukins) on energy metabolism is pivotal, insofar as metabolism drives the differentiation, migration, invasion, and immune evasion in TME, thus facilitating tumor growth and metastasis (51). In the TME, immune cells, including macrophages and lymphocytes, depend on glycolysis (52) and recently some metabolic intermediates have been suggested to be able to regulate IL-1β and IL-6, e.g., succinate, that stabilize hypoxia-inducible factor-1α (HIF-1α) driving IL-1β and IL-6 production (53). Another point is the glycolytic enzyme PKM2 that also controls HIF-1α and thus IL-1β and IL-6 induction (53,54). This scenario is mechanistically dependent on glycolytic activity and glucose-induced reactive oxygen species (ROS) production by the mitochondria, while molecular analyses linked mitochondrial overload to excessive production of ROS. ROS production within macrophages promotes the dimerization of the glycolytic redox-sensing enzyme PKM2 (54). Dimeric PKM2 acts as a protein kinase and phosphorylates the transcription factor STAT3. Phosphorylated STAT3 (PSTAT3) directly increases IL-1β and IL-6 transcription. Nuclear PKM2 specifically functions as a regulator of cytokine production, as basic metabolic regulators affect cellular behavior directly (54,55).

TKTL1 seems to be the key enzyme in a recently described metabolic pathway that binds to the pentoses-phosphate pathway and links the anaerobic degradation of glucose to the production of fatty acids via production of acetyl-CoA (56). In the glycolytic pathway, the activation of the TKTL1 gene leads to oxygen-independent metabolism, increasing glucose intake and lactic acid formation. In addition, it contributes to proliferation, repression of the immune system, angiogenesis, invasion, metastasis, and resistance to treatment (57). The expression of the TKTL1 protein is significantly correlated with increased tumor size, invasion, lymph node metastasis, and TNM stage in gastric cancer (58).

This review revealed that high expressions of HMGCS2 and AldoC are associated with a poor prognosis in OSCC. HMGCS2 is an enzyme involved in both the conversion of fatty acids into carbon derivatives and ketogenesis (59). Ketone bodies may be vital fuel in ketogenesis for tumor initiation or metastasis. Therefore, ketone bodies are a potential high energy resource that can enable a tumor to grow even when cut off from a blood supply. Mitochondrial HMGCS2, the rate-limiting enzyme, catalyzes the first reaction in ketogenesis. Chen et al. (12) found that HMGCS2 enhances invasion and metastasis via activation of the Src signaling pathway through interaction with PPARα into OSCC cells. When Src is activated, it not only induces cancer cell growth and survival, but also promotes the reorganization of the actin cytoskeleton to invade and reduce cell-cell and cell-matrix adhesion, which ultimately further facilitates motility and invasion (12). Patients with OSCC and low HMGCS2 expression seem to survive significantly longer than those with a high HMGCS2 expression level (12).

High expression of AldoC, an enzyme that participates in the conversion of fructose-1,6-bisphosphate into glyceraldehyde-3-phosphate and dihydroxyacetone phosphate, is associated with migration and invasion, and its expression is associated with prognosis and cell migration (17).

Different tumors and tumor cell populations use glucose in different ways. The availability of nutrients determines that some cells in some tumors are predominantly glycolytic, while others primarily have a metabolic phenotype for oxidative phosphorylation (1,6). In the studies reviewed here, an increased glucose uptake, identified using PET-CT, was correlated with a poor prognosis (Figure 2B).

Data about age, tumor type, location, and TNM, as well as the methods used to evaluate metabolic markers, were different in the studies included in this review, which precluded a definition of which marker might be the best choice to establish a prognosis, an interesting question that should be investigated in future studies. However, in general, metabolic changes were associated with a poor survival prognosis (Figure 5).

The data selected to compose the meta-analysis may be influenced by the publication bias. Publication bias means the tendency for published results to be systematically different from the reality. Analysis of clinical trials with a protocol registered in the ClinicalTrial.gov registry base revealed that less than 70% of studies are published (60). Failure to publish results may be due to the author’s decision or study sponsor, who do not submit unfavorable findings for publication, or scientific journals, which may not be interested in disclosing negative results (without statistical significance).

In systematic reviews, the presence of this bias can be identified by means of a funnel and statistical tests (Table 1; Figure 4). The use of these techniques is based on questions of estimation and precision. Inaccurate studies, generally carried out with small sample sizes, may find positive or negative results (statistically significant or not) due to random chances. To acquire the data of more precise studies, we tried contacting the authors without success and this situation obligated us to include small studies (small N) in the meta-analysis (Figures 3 and 4; Table 1).

Moreover, several primary studies that investigated prognosis and overall survival lacked important data, such as odds ratio, hazard ratio, relative risks, and respective confidence intervals. We must take into account that in systematic reviews with meta-analysis, those data are pivotal for a precise conclusion. To solve this problem there are two options: sending e-mails to the authors asking for the raw data or calculate the hazard ratios and the 95%CI from Kaplan-Meier curves. Regarding e-mails, several times the authors did not respond, which makes it very difficult to perform precise analyses. Our suggestion, therefore, is that studies about prognosis and survival should all show descriptive statistics as discussed before.

### Conclusions

Most elements of tumor energy metabolism included in this review were important survival predictors for patients with OSCC. In the past, research used several characteristics of cancer models, such as sustained proliferative signs, evasion of growth suppressors, resistance to cell death, replicative immortality, induction of angiogenesis, and activation of invasion and metastasis. With the development of cancer research, an important feature has emerged: the deregulation of cell energy metabolism, which may bring significant advances to treatments. This systematic review evaluated whether some markers associated with changes in glycolysis, pentose phosphate pathway, and lipid metabolism may be useful when using IHC or RT-PCR findings. We found that high expression levels of GLUT1, pyruvate kinase isoform M2, lactate dehydrogenase isoform 5, TKTL1, and HMGCS2 are associated with poor outcome in OSCC. Further studies should focus on the role of metabolic factors in cell behavior as differentiation, migration, and metastasis with the findings being important to improve new adjuvant treatments and develop prognostic biomarkers.

## Supplementary Material

Click here to view [pdf].
